# Impact of Acute Cardiac Complications After Subarachnoid Hemorrhage on Long-Term Mortality and Cardiovascular Events

**DOI:** 10.1007/s12028-018-0558-0

**Published:** 2018-06-11

**Authors:** Erik Norberg, Helena Odenstedt-Herges, Bertil Rydenhag, Jonatan Oras

**Affiliations:** 1 The Department of Anaesthesiology and Intensive Care Medicine, Institute of Clinical Sciences, Sahlgrenska Academy, University of Gothenburg, Blå Stråket 5, 413 45 Gothenburg, Sweden; 2 The Department of Clinical Neuroscience, Institute of Neuroscience and Physiology, Sahlgrenska Academy, University of Gothenburg, Gothenburg, Sweden

**Keywords:** Subarachnoid hemorrhage, Stroke, Myocardial ischemia, Stress cardiomyopathy

## Abstract

**Background:**

Cardiac complications frequently occur after subarachnoid hemorrhage (SAH) and are associated with an increased risk of neurological complications and poor outcomes. The aim of this study was to evaluate the impact of acute cardiac complications after SAH on long-term mortality and cardiovascular events.

**Methods:**

All patients admitted to our Neuro intensive care unit with verified SAH from January 2010 to April 2015, and electrocardiogram, echocardiogram, and troponin T or NTproBNP data obtained within 72 h of admission were included in the study. Mortality data were obtained from the Swedish population register. Data regarding cause of death and hospitalization for cardiovascular events were obtained from the Swedish Board of Health and Welfare.

**Results:**

A total of 455 patients were included in the study analysis. There were 102 deaths during the study period. Cardiac troponin release (HR 1.08, CI 1.02–1.15 per 100 ng/l, *p* = 0.019), NTproBNP (HR 1.05, CI 1.01–1.09 per 1000 ng/l, *p* = 0.018), and ST-T abnormalities (HR 1.53, CI 1.02–2.29, *p* = 0.040) were independently associated with an increased risk of death. However, these associations were significant only during the first 3 months after the hemorrhage. Cardiac events were observed in 25 patients, and cerebrovascular events were observed in 62 patients during the study period. ST-T abnormalities were independently associated with an increased risk of cardiac events (HR 5.52, CI 2.07–14.7, *p* < 0.001), and stress cardiomyopathy was independently associated with an increased risk of cerebrovascular events (HR 3.65, CI 1.55–8.58, *p* = 0.003).

**Conclusion:**

Cardiac complications after SAH are associated with an increased risk of short-term death. Patients with electrocardiogram abnormalities and stress cardiomyopathy need appropriate follow-up for the identification of cardiac disease or risk factors for cardiovascular disease.

**Electronic supplementary material:**

The online version of this article (10.1007/s12028-018-0558-0) contains supplementary material, which is available to authorized users.

## Background

Acute cardiac complications frequently occur after subarachnoid hemorrhage (SAH). These complications include electrocardiogram (ECG) abnormalities, the release of cardiac biomarkers, and the development of acute stress-induced heart failure resembling Takotsubo cardiomyopathy [1–6]. Cardiac complications after SAH are associated with a complex clinical course and an increased risk of neurological complications and have an increased risk of poor functional outcomes in short- and long-term follow-ups [5–10]. Cardiac complications after SAH are not caused by occlusive coronary artery disease; they are most likely the result of the extensive catecholamine release seen with bleeding [11–14].

Traditionally, SAH patients were believed to have a good prognosis if they recovered from the acute phase of the disease. However, later studies have shown that patients with SAH have higher mortality and a higher risk of cardiac and cerebrovascular events than the general population [15–17]. Increasing evidence indicates that minor, subclinical troponin release and stress cardiomyopathy are associated with an increased risk of death and adverse cardiovascular outcomes in other patient categories [18–21]. Therefore, SAH patients presenting with cardiac complications are potentially at high risk for later cardiovascular events but these associations have barely been studied [22, 23].

The aim of this study was to evaluate the impact of acute cardiac complications after SAH on long-term mortality and cardiac and cerebrovascular events.

## Methods

This is a retrospective, single-center study. The study was approved by the regional ethics committee at the University of Gothenburg.

### Patient Inclusion

All patients admitted to our neuro-intensive care unit (NICU) between January 1, 2010 and April 30, 2015 with suspected or verified spontaneous, non-traumatic SAH were eligible for inclusion in the study. Patients were identified by having the admission diagnosis SAH in our local ICU register. The SAH diagnosis was verified by computer tomography or lumbar puncture. Patients with an ECG, echocardiography, or measurements of any of the cardiac biomarkers, including high-sensitive troponin T (hsTnT) or N-terminal pro-B-type natriuretic peptide (NTproBNP), obtained within 72 h after symptom onset were included in the study. Patients with benign bleeding (i.e., prepontine) were excluded from the study. Since January 2010, we have focused on cardiac complications after SAH, and since January 2012, we have routinely obtained ECG, hsTnT, and NTproBNP data in all patients admitted to our NICU on admission and the following 3 days. We liberally perform echocardiography based on the clinical condition and cardiac biomarker release, which has been shown to have an association with stress cardiomyopathy [24]. All patients were treated in accordance with a local protocol consistent with the strategies given by the Neurocritical Care consensus conference and American Heart Association guidelines [25, 26].

### Data Collection

The following data were retrieved from medical records: medical history associated with risk factors for cardiovascular disease, findings on ECG on admission, findings on echocardiography within 72 h from admission, and peak levels of hsTnT and NTproBNP within 72 h from admission. The severity of SAH was graded clinically with World Federation of Neurosurgery Scale (WFNS) scores by the attending neurosurgeon upon admission to the NICU and radiographically using the modified Fisher scale [27, 28]. The presence of an aneurysm, aneurysm treatment, and the presence of an intracerebral hematoma were noted. A new computed tomography-verified cerebral infarction from onset of symptoms up to 6 weeks after SAH was noted as a cerebral infarction. All data collection was performed before registry data were obtained.

### Analyses and Definitions

hsTnT was analyzed with the Roche high-sensitive troponin T assay with a coefficient of variation of 3.4%. NTproBNP was analyzed with the Elecsys^®^ assay (Roche) on the Cobas platform with a coefficient of variation of 3.8%, as reported by our hospital laboratory. Left ventricular (LV) ejection fraction was assessed with the Simpson biplane method. Sixteen LV segments were individually evaluated and scored on the basis of motion and thickening as follows: score 1 (normokinesia), score 2 (hypokinesia), score 3 (akinesia), and score 4 (dyskinesia). Stress cardiomyopathy was defined as (1) the presence of regional wall motion abnormalities (RWMA) extending over more than one typical coronary artery distribution, (2) verified regression of RWMA, (3) modest elevation of troponins (out of proportion for the extent of the RWMA), (4) no history of coronary artery disease and (5) no other credible explanation for the RWMA, for example, ischemia, tachycardia, or viral myocarditis. This definition was based on the criteria for Takotsubo syndrome from the European Society of Cardiology [29]. ECG interpretation was performed according to the Minnesota code [30]. ECG abnormalities were defined as follows: ST elevation, an elevation at the J-point in two contiguous leads of ≥ 0.2 mV in V2-V3 and ≥ 0.1 mV in other leads; ST depression, horizontal or down-sloping depression ≥ 0.05 mV in two contiguous leads; T-wave inversion, a negative T-wave ≥ 0.1 mV in two contiguous leads; long QT, a corrected QT time ≥ 440 ms in men and ≥ 460 ms in women; u-wave, > 2 mm or 25% of the T-wave; left bundle branch block (LBBB), QRS duration > 120 ms, a dominant S-wave in V1, and a broad monophasic R-wave with the absence of the Q-wave in lateral leads; right bundle branch block (RBBB), QRS duration > 120 ms, RSR´ pattern in V1–V3 and a wide slurred S-wave in the lateral leads.

### Registry Data

Mortality data and the date of death were obtained from the Swedish population register. The mortality data were obtained until December 31, 2016. The cause of death was obtained from the register of cause of death maintained by the Swedish National Board of Health and Welfare (Dödsorsaksregistret, Socialstyrelsen). This register prospectively collects data regarding causes of death among all Swedish inhabitants. The data for cardiac and cerebrovascular events after SAH were obtained from the Swedish National Patient Register at the National Board of Health and Welfare (Patientregistret, Socialstyrelsen). This register prospectively collects data regarding primary and secondary discharge diagnoses and covers all inpatient care in Sweden. These data were obtained as ICD-10 diagnoses. The data from the Swedish National Board of Health and Welfare were obtained until December 31, 2015. A cardiac event was defined as any of the following death or hospital discharge ICD-10 codes at an admission after primary SAH discharge: I20–I25, I40–I43, I46, and I50 (i.e., diagnoses including cardiac ischemia, cardiomyopathy, cardiac arrest, and heart failure, respectively). A cerebrovascular event was defined as having any of the following death or hospital discharge ICD-10 codes at an admission after primary SAH discharge: I61–I67 (i.e., diagnoses including non-traumatic intracerebral hemorrhage, cerebral infarction, and cerebral atherosclerosis, respectively). Cerebrovascular events occurring within six weeks after SAH were attributed to delayed cerebral ischemia and were not registered as an event in the present study [31].

### Statistics

All continuous variables were assessed for normality. Normally distributed data are presented as the mean ± standard deviation, and non-normally distributed data are presented as the median (interquartile range). Student’s *t* test was used to compare the means between two groups with normally distributed variables, and the Mann–Whiney *U* test was used to compare the medians between two groups with non-normally distributed variables. Fisher’s exact test was used to compare the distributions of dichotomous variables with binary outcomes. Kaplan–Meier curves with the log-rank test were used to compare incidences and survival over time for dichotomous variables. Cox regression was used to compare incidences and survival over time in multivariable analyses. For identification of baseline risk factors associated with an increased risk of death and cardiovascular events over time, a multivariable analysis with a forward modeling approach was used. The variables with the lowest *p* values were consecutively included, and variables with a *p* value < 0.05 were retained in the model. The cardiac variables (ECG, stress cardiomyopathy, peak levels of hsTnT, NTproBNP) were adjusted for the risk factors identified. A *p* value < 0.05 was considered significant. IBM SPSS 24.0 was used for the statistical analyses.

## Results

### Study Cohort

**Fig. 1:**
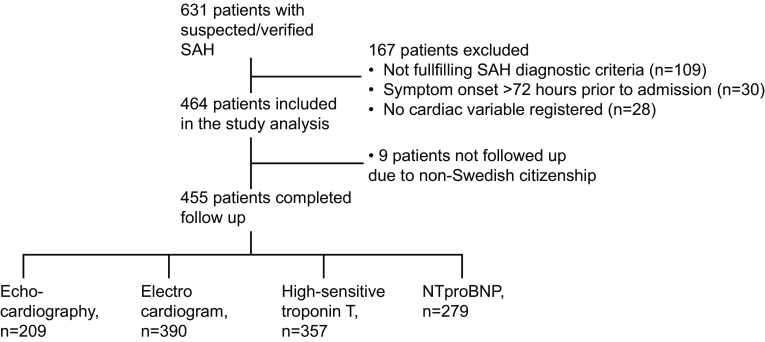
Study flow chart. NTproBNP, N-terminal pro-B-type natriuretic peptide; SAH, subarachnoid hemorrhage

The study flowchart is shown in Fig. 1. During the study period, 634 patients were admitted with suspected or verified SAH. A total of 167 patients were excluded from the analysis because they did not fulfill the SAH criteria, for example, prepontine bleeding (*n* = 109), admission > 72 h after symptom onset (*n* = 30), and no records of ECG, troponin/NTproBNP, or echocardiography (*n* = 28). Of the 464 patients included in the analysis, nine patients were non-Swedish citizens, so follow-up data were not retrievable. Of the 455 patients included in the analysis, most patients (88%) were admitted within 24 h of symptom onset, 8% of the patients were admitted on day 2 after symptom onset, and the remaining 4% were admitted on day 3 after symptom onset.

### Baseline Data

All baseline data are shown in Table 1. The mean age of the study cohort was 58 years, and 61% of the patients were women. A total of 165 (36%) patients had a history of hypertension, 30 (6%) patients had a history of cardiac disease, and 21 (4%) patients had a history of cerebrovascular disease. High-grade SAH (WFNS 4–5) was observed in 130 (28%) patients, and 193 (42%) patients had modified Fisher grade 4 SAH. An aneurysm was verified in 393 (86%) patients, including 261 who were treated with intravascular embolization, 124 who were treated with surgery, and 20 who were not treated due to poor prognosis or severe comorbidity. A total of 159 (35%) patients had a fulfilled the criteria for a new cerebral infarction.

**Table 1:** Patient characteristics

Category	Variable	Value
Background data	Age, years	58 ± 13
	Female sex, *n* (%)	277 (61)
Medical history	Hypertension, *n* (%)	165 (36)
	Cardiac disease, *n* (%)	30 (6)
	Cerebrovascular disease, *n* (%)	21 (4)
	Previous SAH, *n* (%)	5 (1)
	COPD, *n* (%)	14 (3)
	Malignancy, *n* (%)	21 (4)
	Diabetes, *n* (%)	14 (3)
	Renal disease, *n* (%)	6 (1)
	Other, *n* (%)	31 (7)
Neurological status	WFNS grade 1, *n* (%)	217 (48)
	WFNS grade 2, *n* (%)	93 (20)
	WFNS grade 3, *n* (%)	14 (3)
	WFNS grade 4, *n* (%)	79 (17)
	WFNS grade 5, *n* (%)	51 (11)
Radiological finding	Modified Fisher 1, *n* (%)	68 (15)
	Modified Fisher 2, *n* (%)	80 (18)
	Modified Fisher 3, *n* (%)	114 (25)
	Modified Fisher 4, *n* (%)	193 (42)
	Intracerebral hematoma, *n* (%)	60 (13)
	Verified aneurysm, *n* (%)	393 (86)
	Cerebral infarction due to SAH, *n* (%)	159 (35)
Treatment	Ventricular drainage, *n* (%)	189 (42)
	Embolization, *n* (%)	250 (55)
	Surgery, *n* (%)	119 (26)

*COPD* chronic obstructive pulmonary disease, *SAH* subarachnoid hemorrhage, *WFNS* World Federation of Neurosurgery Scale for grading of SAH

### Cardiac Complications

ECG was recorded in 390 (86%) patients, including 179 with a normal ECG. The most common ECG anomalies were ST-T abnormalities (ST elevation, T-wave inversion, or ST depression), which were found in 134 patients. Echocardiography was performed in 209 (46%) patients, of whom 134 were examined on day 1, 48 were examined on day 2, and 27 were examined on day 3 after onset of symptoms. Stress cardiomyopathy was observed in 31 (15%) patients, who had a median ejection fraction of 45%. Troponin was assessed in 357 (78%) patients, and the median value was 14.7 ng/l. NTproBNP was assessed in 279 (61%) patients, and the median value was 879 ng/l (Table 2). Echocardiography was more frequently performed in patients with high-grade SAH (performed in 62% of the patients presenting with WFNS 4–5 SAH vs. 38% of the patients presenting with WFNS 1–3 SAH). This bias was not evident for ECG recordings or blood sample obtainment for the measurement of troponin or NTproBNP.

**Table 2:** Cardiac variables

Category	Variable	Value
Cardiac biomarkers	hsTnT, ng/l	14.7 (6.5–59.2)
	NTproBNP, ng/l	879 (408–1895)
ECG	Normal, *N* (%)	179 (46)
	ST elevation, *N* (%)	8 (2)
	T-wave inversion, *N* (%)	20 (5)
	ST depression, *N* (%)	106 (27)
	Long QT/u-wave, *N* (%)	36 (9)
	LBBB/RBBB, *N* (%)	19 (5)
	q-wave, *N* (%)	15 (4)
	Arrhythmias, *N* (%)	11 (3)
Echocardiographic	Stress cardiomyopathy, *N* (%)	31 (15)
	EF in non-SCM patients, %	65 (60–65)
	EF in SCM patients, %	45 (35–50)

Continuous variables are presented as the median (interquartile range)

*EF* ejection fraction, *hsTnT* high-sensitive troponin T, *LBBB* left bundle branch block, *NTproBNP* N-terminal pro-B-type natriuretic peptide, *RBBB* right bundle branch block, *SCM* stress cardiomyopathy

### Mortality

There were 102 deaths in the cohort until December 31, 2016. The median time to death was 28 days; 76 (75%) deaths occurred within 3 months, and 87 (85%) deaths occurred within one year after SAH. Data regarding cause of death were available until December 31, 2015 (*n* = 91). Seventy-three of the deaths were due to primary SAH, six deaths were attributed to cerebrovascular disease other than SAH, and two deaths were attributed to acute myocardial infarction. There were 11 non-cardiovascular deaths, including six due to malignancy, three due to pulmonary disease, one due to liver disease, one due to gastrointestinal bleeding, and one due to infectious disease (influenza pneumonia). Age, poor neurological status on admission (WFNS 4–5), and the presence of cerebral infarction during hospitalization were all independently associated with an increased risk of death. A history of either cardiac, cerebrovascular or renal disease was the most significant comorbidity variable associated with an increased risk of death (Supplementary material).

### Cardiac and Cerebrovascular Events

A total of 57 cardiac events were observed during the study period among the 25 patients. The incidence rate was 0.048 per follow-up year. The median time to the first event was 0.57 years. Age, history of cardiac disease, and the male sex were all independent variables associated with an increased risk of cardiac events (Supplementary material). A total of 84 cerebrovascular events were observed during the study period among 62 patients. The incidence rate was 0.071 events per follow-up year, and the median time to the first event was 1.1 years. A history of hypertension was the only variable associated with an increased risk of cerebrovascular events (Supplementary material).

### Impact of Cardiac Complications on Mortality

**Fig. 2:**
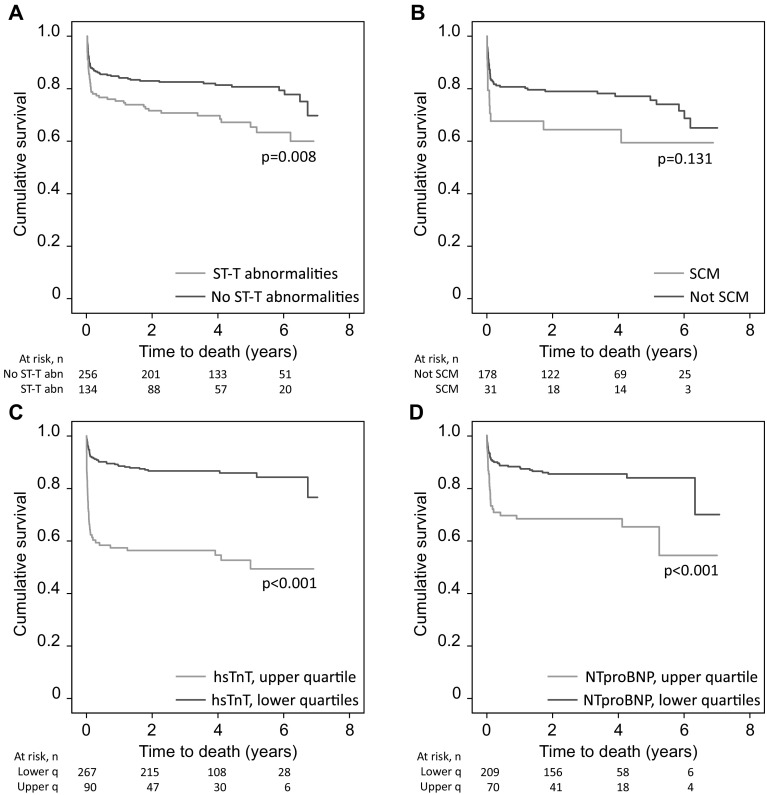
Impact of acute cardiac complications after SAH on mortality. Impact of ST-T abnormalities (**a**), stress cardiomyopathy (**b**), hsTnT (**c**), and NTproBNP (**d**) on mortality. The continuous variables, hsTnT and NTproBNP, were divided into the top quartile (red line) and bottom three quartiles (blue line). The differences between the groups were significant only during the first 3 months after the hemorrhage. hsTnT, high-sensitive troponin T; NTproBNP, N-terminal pro-B-type natriuretic peptide; SCM, stress cardiomyopathy (Color figure online)

The ECG anomaly with the strongest association with the risk of death was ST-T abnormalities (ST elevation, T-wave inversion, or ST depression, *p* = 0.003; Fig. 2a). Peak levels of hsTnT (*p* < 0.001, Fig. 2c) and NTproBNP (*p* < 0.001, Fig. 2d) were associated with a higher risk of death. None of the other ECG changes were associated with an increased risk of death. Patients presenting with stress cardiomyopathy did not have a higher risk of death (*p* = 0.131, Fig. 2b). After adjusting for the most significant baseline variables (age, WFNS 4–5, cerebral infarction, and history of cardiovascular or renal disease), ST-T abnormalities and peak levels of hsTnT and NTproBNP were still associated with an increased risk of death (Table 3). However, these differences were significant only during the first 3 months after hemorrhage but not thereafter (Table 3, Fig. 2a–d).

**Table 3:** Cox regression of cardiac variables associated with the risk of death

Variable	Unadjusted			Adjusted^a^		
	HR	95% CI for HR	*p* value	HR	95% CI for HR	*p* value
*Death, whole study period*						
ST-T abnormalities	1.68	1.13–2.50	0.009	1.53	1.02–2.29	0.040
Stress cardiomyopathy	1.60	0.95–3.23	0.148	1.38	0.73–2.63	0.532
hsTnT, per 100 ng/l	1.17	1.10–1.23	< 0.001	1.08	1.02–1.15	0.019
NTproBNP, per 1000 ng/l	1.08	1.05–1.12	< 0.001	1.05	1.01–1.09	0.018
*Death, later than 3* *months after SAH*						
ST-T abnormalities	1.89	0.87–4.09	0.106	1.77	0.78–4.00	0.171
Stress cardiomyopathy	2.41	0.47–12.5	0.292	6.07	0.71–51.3	0.098
hsTnT, per 100 ng/l	1.02	0.82–1.27	0.836	0.98	0.76–1.26	0.890
NTproBNP, per 1000 ng/l	0.73	0.39–1.37	0.343	0.52	0.22–1.24	0.140

*95% CI* 95% confidence interval, *HR* hazard ratio, *hsTnT* high-sensitive troponin T, *NTproBNP* N-terminal pro-B-type natriuretic peptide

^a^Adjusted for age, WFNS score 4–5, cerebral infarction during hospital stay, and history of cardiac, cerebrovascular, or renal disease

### Impact of Cardiac Complications on Cardiac and Cerebrovascular Events

**Fig. 3:**
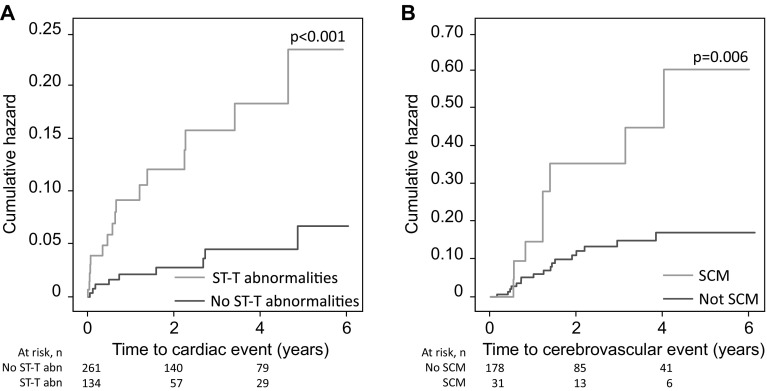
Impact of acute cardiac complications after SAH on cardiovascular events. Impacts of ST-T abnormalities on cardiac events (**a**) and stress cardiomyopathy on cerebrovascular events (**b**). None of the other cardiac variables were associated with an increased risk of cardiac or cerebrovascular events. SCM, stress cardiomyopathy

ST-T abnormalities were associated with an increased risk of cardiac events (*p* < 0.001, Fig. 3a). This finding remained significant after adjusting for age, a history of cardiac disease, and sex (*p* < 0.001, Table 4). Neither peak levels of troponin and NTproBNP nor the presence of stress cardiomyopathy was associated with an increased risk of cardiac events (Table 4).

**Table 4:** Cox regression of cardiac variables associated with later cardiac and cerebrovascular events

Variable	Unadjusted			Adjusted		
	HR	95% CI for HR	*p* value	HR	95% CI for HR	*p* value
*Cardiac events*						
ST-T abnormalities	5.72	2.21–14.7	< 0.001	5.52	2.07–14.7	< 0.001
Stress cardiomyopathy	0.51	0.07–3.93	0.521	0.87	0.10–7.75	0.904
hsTnT, per 100 ng/l	0.90	0.66–1.24	0.522	0.93	0.68–1.27	0.650
NTproBNP, per 1000 ng/l	0.86	0.58–1.28	0.464	0.73	0.41–1.28	0.268
*Cerebrovascular events*						
ST-T abnormalities	0.97	0.54–1.74	0.912	0.95	0.53–1.72	0.871
Stress cardiomyopathy	3.09	1.33–7.16	0.009	3.65	1.55–8.58	0.003
hsTnT, per 100 ng/l	0.98	0.83–1.15	0.780	0.99	0.85–1.15	0.863
NTproBNP, per 1000 ng/l	1.07	0.97–1.17	0.191	1.03	0.93–1.14	0.578

Cardiac events were adjusted for age, sex, and history of cardiovascular disease. Cerebrovascular events were adjusted for history of hypertension

*95% CI* 95% confidence interval, *HR* hazard ratio, *hsTnT* high-sensitive troponin T, *NTproBNP* N-terminal pro-B-type natriuretic peptide

Stress cardiomyopathy was associated with an increased risk of cerebrovascular events (*p* = 0.006, Fig. 3b). This finding remained significant after adjusting for a history of hypertension (*p* = 0.003, Table 4). Peak levels of troponin and NTproBNP and ST-T abnormalities were not associated with an increased risk of cerebrovascular events (Table 4).

## Conclusion

The main findings of this study were that ST-T abnormalities and peak levels of troponin and NTproBNP within 72 h after the onset of SAH were associated with an increased risk of death but only during the first three months after the event. Furthermore, in SAH patients surviving hospital stay, ST-T abnormalities were associated with an increased risk of cardiac events, and stress cardiomyopathy was associated with an increased risk of cerebrovascular events.

Cardiac complications after SAH are well described in the literature and occur in up to 50% of SAH patients [1–6]. Several studies have shown that such cardiac complications are independently associated with an increased risk of delayed cerebral ischemia, cerebral infarction, and poor outcomes [5–10]. SAH patients are also at a higher risk of long-term death and cerebrovascular disease than the general population [15–17]. Furthermore, studies have shown that minor troponin release and stress cardiomyopathy, conditions believed to have a good prognosis, carry a high risk of death and later cardiovascular events [18–21]. Our hypothesis was that patients with cardiac complications after SAH are high-risk patients for cardiovascular disease with an increased risk of death. We found that cardiac biomarker release and ST-T abnormalities were independent risk factors for an increased risk of death also after adjusting for important factors such as age, neurological status on admission, and cerebral infarction. Stress cardiomyopathy was not associated with an increased risk of death, but this result should be interpreted with caution because patients who underwent echocardiography had more severe disease and higher overall mortality. In contrast to our hypothesis, the increased risk of death was significant only shortly after SAH, i.e., within the first 3 months after hemorrhage. Nevertheless, our results support the importance of cardiac complications in the acute care of SAH patients.

Cardiac complications after SAH are not due to coronary artery disease; they are most likely a consequence of sympathetic overstimulation [5–7]. In the present study, we found that ST-T abnormalities were an independent risk factor for cardiac events. This association was not evident for any of the other cardiac variables. Epidemiological studies of healthy subjects have shown that patients with ST-T abnormalities, including minor ST-T abnormalities, are at a higher risk of later cardiac events, suggesting that these patients may have undetected cardiac disease. [32, 33]. ST-T abnormalities after SAH are frequent and are most likely due to sympathetic stress after SAH, which could easily be ignored and regarded as unspecific findings. However, the present study suggests that some of these patients may suffer from undetected cardiac disease. It is possible that such disease is revealed on ECG due to the cardiac stress afflicting the heart after SAH.

We found an association between stress cardiomyopathy and an increased risk of cerebrovascular events. These events were not a direct consequence of SAH because all insults occurring within six weeks from the hemorrhage were excluded. Studies have shown a risk of thromboembolic events of 4–12% in the acute phase of stress cardiomyopathy in other populations, most likely due to the risk of cardiac thrombus formation in the akinetic left ventricle [29, 34–36]. In patients with myocardial infarction, a cardiac thrombus may embolize months or years after the infarction [37, 38]. Unfortunately, long-term data regarding ventricular thrombosis and embolization after stress cardiomyopathy are lacking. It is possible that some of the patients with stress cardiomyopathy and cerebral insults in the present study could have had an undetected cardiac thrombus. Furthermore, studies have shown that patients with stress cardiomyopathy very frequently have risk factors for cerebrovascular disease, such as hypertension, hyperlipidaemia, and diabetes [20, 39]. The increased incidence of cerebral insults may also be due to undetected and untreated risk factors of cerebrovascular disease.

Based on the results of the present study, we recommend that ECG should be routinely recoded in all patients with SAH. Echocardiographic examination for detection of stress cardiomyopathy is desirable in all patients and should, at the very least, be performed in patients with high-grade SAH or significant increase in troponin and NTproBNP levels [24]. Intensive hemodynamic monitoring with goal-directed hemodynamic therapy might be beneficial in SAH patients with DCI and stress cardiomyopathy [40]. Patients with ST-T abnormalities and stress cardiomyopathy should receive a diagnostic workup for cardiac disease or risk factors for cerebrovascular disease. Furthermore, patients with stress cardiomyopathy should have a late follow-up echo to exclude ventricular thrombus formation. It is important for ICU physicians to be aware of such findings, since these abnormalities are frequently seen in the ICU, to be able to establish an appropriate follow-up plan for patients.

To the best of our knowledge, there are only two studies on long-term complications and cardiac events after SAH [22, 23]. Consistent with the present study, they revealed an increased risk of death, especially early on after SAH, and an increased risk of cardiac events. One of these studies was limited by its design as a registry study, with no detailed data regarding which cardiac complications affected each SAH patient [22]. The other study reported on only the association between stress cardiomyopathy and death [23].

The main limitation of the present study is the relatively short follow-up time. It is also a single-center study. Furthermore, there was a bias in having echocardiography performed in patients with more severe disease. We did not have a matched non-SAH population with which to compare incidences of cardiac and cerebrovascular events, but such a comparison has been reported previously and was not the aim of the current study. The strength of this study is that we had detailed data, including follow-up data, for all included patients. We also studied a recent cohort, and the majority of the patients were treated according to current guidelines.

In conclusion, acute cardiac complications after SAH are associated with an increased risk of short-term death. Furthermore, ST-T abnormalities and stress cardiomyopathy were associated with an increased risk of cardiac and cerebrovascular events. These cardiac complications should not be ignored by clinicians and require accurate follow-up for the identification of undetected cardiac thrombus, cardiac disease, or risk factors for cerebrovascular disease.

## Electronic supplementary material

Below is the link to the electronic supplementary material.

Supplementary material 1 (DOCX 21 kb)
