# Supplementary material for: Impact of Acute Cardiac Complications After Subarachnoid Hemorrhage on Long-Term Mortality and Cardiovascular Events
Source: Neurocrit Care. 2018 Jun 11;29(3):404–12. doi: 10.1007/s12028-018-0558-0 (PMC6290719; doi:10.1007/s12028-018-0558-0)
Supplement: Supplementary file 1 — Supplementary material 1 (DOCX 21 kb) [file 12028_2018_558_MOESM1_ESM.docx]

| **Univariable regression analysis of baseline variables associated with an increased risk of death** | | | | |
| --- | --- | --- | --- | --- |
| Category | Variable | HR | 95% CI | p-value |
| Background data | Age | 1.06 | 1.04 - 1.08 | <0.001 |
|  | Sex | 1.37 | 0.94 - 2.02 | 0.105 |
| Medical history | Hypertension | 1.74 | 1.18 - 2.57 | 0.005 |
|  | Cardiac disease | 2.31 | 1.26 - 4.22 | 0.006 |
|  | Cerebravascular disease | 2.35 | 1.19 - 4.66 | 0.014 |
|  | Previous SAH | 2.50 | 0.79 - 7.91 | 0.118 |
|  | COPD | 1.19 | 0.44 - 3.24 | 0.730 |
|  | Cancer | 1.87 | 0.91 - 3.85 | 0.088 |
|  | Diabetes | 1.36 | 0.50 - 3.71 | 0.544 |
|  | Renal disease | 3.25 | 1.18 - 8.96 | 0.022 |
|  | CVD or renal disease | 2.32 | 1.42 - 3.77 | 0.001 |
| Neurological finding | WFNS 4-5 | 3.24 | 2.21 - 4.76 | <0.001 |
| Radiological finding | Modified Fischer grade 4 | 2.44 | 1.64 - 3.61 | <0.001 |
|  | Intracerebral hemorrhage | 1.76 | 1.09 - 2.84 | 0.021 |
|  | Verified aneurysm | 0.92 | 0.73 - 1.16 | 0.464 |
|  | Cerebral infarction | 3.07 | 2.08 - 4.55 | <0.001 |
| Treatment | Embolization vs surgery | 0.66 | 0.39 - 1.12 | 0.127 |

HR, Hazard ratio; CI, confidence intervall for hazard ratio; SAH, subarachnoid hemorrhage; COPD, chronic obstructive pulmonary disease; CVD, cardiovascular disease; WFNS, World Federation of Neurosurgery Scale for grading of SAH. The combination of having any of cardiac-, cerebrovascular-, or renal disease were most important of the medical history-variables and were merged to one variable for the multivariable analysis.

| **Final multivariable regression analysis of variables associated with an increased risk of death** | | | |
| --- | --- | --- | --- |
| Variable | HR | 95% CI | p-value |
| Age | 1.06 | 1.04 - 1.08 | <0.001 |
| WFNS grade 4-5 | 2.58 | 1.73 - 3.85 | <0.001 |
| Cerebral infarction | 2.27 | 1.51 - 3.42 | <0.001 |
| History of CVD or renal disease | 2.24 | 1.03 - 4.84 | 0.041 |

HR, Hazard ratio; CI, confidence intervall for hazard ratio; COPD, chronic obstructive pulmonary disease; CVD, cardiovascular disease; WFNS, World Federation of Neurosurgery Scale for grading of SAH.

| **Univariable regression analysis of baseline variables associated with an increased risk of cardiac events** | | | | |
| --- | --- | --- | --- | --- |
| Category | Varable | HR | 95% CI | p-value |
| Background data | Age | 1.10 | 1.06 - 1.14 | <0.001 |
|  | Sex | 2.64 | 1.24 - 5.64 | 0.012 |
| Medical history | Hypertension | 2.43 | 1.15 - 5.10 | 0.020 |
|  | Cardiac disease | 12.29 | 5.47 - 27.59 | <0.001 |
|  | Cerebrovascular disease | 4.81 | 1.66 - 13.91 | 0.004 |
|  | Previous SAH | 2.97 | 0.40 - 21.97 | 0.286 |
|  | COPD | 1.14 | 0.16 - 8.40 | 0.897 |
|  | Cancer | 0.84 | 0.11 - 6.22 | 0.868 |
|  | Diabetes | - | - | - |
|  | Renal disease | - | - | - |
| Neurological status | WFNS grade 4-5 | 1.10 | 0.47 - 2.58 | 0.832 |
| Radiological finding | Modified Fischer grade 4 | 0.65 | 0.28 - 1.47 | 0.298 |
|  | Intracerebral hemorrhage | 1.14 | 0.40 - 3.30 | 0.806 |
|  | Verified aneurysm | 0.85 | 0.53 - 1.35 | 0.484 |
|  | Cerebral infarcion | 1.88 | 0.89 - 3.97 | 0.096 |
| Treatment | Embolization vs surgery | 0.80 | 0.33 - 1.95 | 0.626 |

HR, Hazard ratio; CI, confidence intervall for hazard ratio; SAH, subarachnoid hemorrhage; COPD, chronic obstructive pulmonary disease; WFNS, World Federation of Neurosurgery Scale for grading of SAH. There were too few cases to make an adequate regression model for diabetes and renal disease.

| **Final multivariable regression analysis of variables associated with an increased risk of death** | | | |
| --- | --- | --- | --- |
| Variable | HR | 95% CI | p-value |
| Age | 1.10 | 1.05 - 1.14 | <0.001 |
| Sex | 3.14 | 1.44 - 6.83 | 0.004 |
| History of cardiac disease | 6.49 | 2.82 - 14.91 | <0.001 |

HR, Hazard ratio; CI, confidence intervall for hazard ratio

| **Univariable regression analysis of baseline variables associated with an increased risk of cerebrovascular events** | | | | |
| --- | --- | --- | --- | --- |
| Category | Variable | HR | 95% CI | p-value |
| Background variables | Age | 1.01 | 0.99 - 1.04 | 0.167 |
|  | Sex | 1.13 | 0.68 - 1.87 | 0.644 |
| Medical history | Hypertension | 2.12 | 1.28 - 3.50 | 0.003 |
|  | Cardiac disease | 2.45 | 0.98 - 6.11 | 0.055 |
|  | Cerebrovascular disease | 2.00 | 0.73 - 5.52 | 0.180 |
|  | Previous SAH | 1.24 | 0.17 - 8.97 | 0.830 |
|  | COPD | 1.58 | 0.50 - 5.05 | 0.438 |
|  | Cancer | 1.61 | 0.58 - 4.43 | 0.358 |
|  | Diabetes | - | - | - |
|  | Renal disease | - | - | - |
| Neurological status | WFNS grade 4-5 | 1.31 | 0.75 - 2.28 | 0.344 |
| Radiological finding | Modified Fischer grade 4 | 0.90 | 0.54 - 1.51 | 0.686 |
|  | Intracerebral hemorrhage | 0.47 | 0.17 - 1.28 | 0.139 |
|  | Verified aneurysm | 1.86 | 0.88 - 3.91 | 0.102 |
|  | Cerebral infarction | 1.15 | 0.68 - 1.94 | 0.607 |
| Treatment | Embolization vs surgery | 1.21 | 0.72 - 2.05 | 0.473 |

HR, Hazard ratio; CI, confidence intervall for hazard ratio; SAH, subarachnoid hemorrhage; COPD, chronic obstructive pulmonary disease; CVD, cardiovascular disease; WFNS, World Federation of Neurosurgery Scale for grading of SAH. There were too few cases to make an adequate regression model for diabetes and renal disease.

| **Final multivariable regression analysis of variables associated with an increased risk of cerebrovascular events** | | | |
| --- | --- | --- | --- |
| Variable | HR | 95% CI | p-value |
| History of hypertension | 2.12 | 1.28 - 3.5 | 0.003 |

HR, Hazard ratio; CI, confidence intervall for hazard ratio
